# Aflibercept Traps Galectin-1, an Angiogenic Factor Associated with Diabetic Retinopathy

**DOI:** 10.1038/srep17946

**Published:** 2015-12-09

**Authors:** Atsuhiro Kanda, Kousuke Noda, Wataru Saito, Susumu Ishida

**Affiliations:** 1 Laboratory of Ocular Cell Biology and Visual Science, Hokkaido University Graduate School of Medicine, Sapporo, Hokkaido 060-8638, Japan; 2 Department of Ophthalmology, Hokkaido University Graduate School of Medicine, Sapporo, Hokkaido 060-8638, Japan

## Abstract

Vascular endothelial growth factor (VEGF)-A-driven angiogenesis contributes to various disorders including cancer and proliferative diabetic retinopathy (PDR). Among several VEGF-A blockers clinically used is aflibercept, a chimeric VEGFR1/VEGFR2-based decoy receptor fused to the Fc fragment of IgG1 (*i.e.*, VEGFR1/VEGFR2-Fc). Here, we revealed a novel anti-angiogenic function for aflibercept beyond its antagonism against VEGF family members. Immunoprecipitation and mass spectrometry analyses identified galectin-1 as an aflibercept-interacting protein. Biolayer interferometry revealed aflibercept binding to galectin-1 with higher affinity than VEGFR1-Fc and VEGFR2-Fc, which was abolished by deglycosylation of aflibercept with peptide:*N*-glycosidase F. Retinal *LGALS1/Galectin-1* mRNA expression was enhanced *in vitro* by hypoxic stimulation and *in vivo* by induction of diseases including diabetes. Galectin-1 immunoreactivity co-localized with VEGFR2 in neovascular tissues surgically excised from human eyes with PDR. Compared with non-diabetic controls, intravitreal galectin-1 protein levels were elevated in PDR eyes, showing no correlation with increased VEGF-A levels. Preoperative injection of bevacizumab, a monoclonal antibody to VEGF-A, reduced the VEGF-A, but not galectin-1, levels. Galectin-1 application to human retinal microvascular endothelial cells up-regulated VEGFR2 phosphorylation, which was eliminated by aflibercept. Our present findings demonstrated the neutralizing efficacy of aflibercept against galectin-1, an angiogenic factor associated with PDR independently of VEGF-A.

Angiogenesis is the formation of new blood vessels from a pre-existing vascular bed and usually occurs during embryogenesis, wound healing, and organ regeneration. This biological phenomenon, executed by vascular endothelial cells that constitute the inner lining of blood vessels, comprises multistep programs including matrix degradation, cell proliferation/migration, tube formation and matrix deposition, and eventually results in the formation of a new microvasculature. Numerous studies have been conducted to show that vascular endothelial growth factor (VEGF)-A is a major endothelial cell mitogen among a rich variety of pro-angiogenic molecules reported so far.

Tumor growth and metastasis depend on angiogenesis activated by pathological signaling from tumor cells^1^. Activation of the VEGF-A-VEGFR2 (the Flk-1/KDR receptor) axis initiates a network of multiple signaling processes, which has been implicated as a key pathway required for pathological angiogenesis in cancer^2^. Pathological angiogenesis is also a hallmark of various ischemic and inflammatory disorders^3^. In the field of ophthalmology, abnormal angiogenesis driven by VEGF-A was shown to cause vision-threatening retinal and choroidal diseases including proliferative diabetic retinopathy (PDR) and age-related macular degeneration (AMD)^4^,^5^. PDR, the advanced stage of diabetic retinopathy, is characterized by fibrovascular proliferation, in which fibrovascular tissue is formed by the extension of retinal new vessels into the vitreous cavity, resulting in severe complications such as vitreous hemorrhage and tractional retinal detachment. Wet type of AMD is complicated by choroidal neovascularization (CNV) involving the macula, the photoreceptor-dense central area of the retina, and thus causing central vision loss and blindness with submacular hemorrhage and exudative retinal detachment. Several anti-VEGF-A agents, successfully developed, have so far been widely used for the treatment of cancer and eye diseases^6^,^7^,^8^,^9^,^10^,^11^.

Aflibercept, also known as VEGF-Trap, is currently used for the treatment of various ocular abnormalities as Eylea (i.e., CNV seen in wet AMD^10^ and pathological myopia^12^, and macular edema seen in branch and central retinal vein occlusions^13^,^14^,^15^ and diabetic retinopathy^9^) and metastatic colorectal cancer as Zaltrap^11^. Aflibercept is a pharmacologically engineered glycoprotein consisting of the ligand-binding elements of human VEGFR1 (Ig domain 2) and VEGFR2 (Ig domain 3), fused to the Fc portion of human IgG1^16^. This unique protein structure allows aflibercept to bind VEGF-A with higher affinity than other VEGF-A blockers and to interact with other VEGF family members such as VEGF-B and placental growth factor^17^; however, its biological potential has not been completely unveiled. Due to its structure artificially designed and thus naturally non-existing, we hypothesized a possibility that aflibercept may interact with an unexpected protein so as to modify its function. Here we report the first evidence that aflibercept binds and neutralizes a pro-angiogenic molecule outside of the VEGF family, showing an additional anti-angiogenic capability beyond its originally prepared anti-VEGF function.

## Results

### Identification of aflibercept-interacting proteins

**Figure 1:**
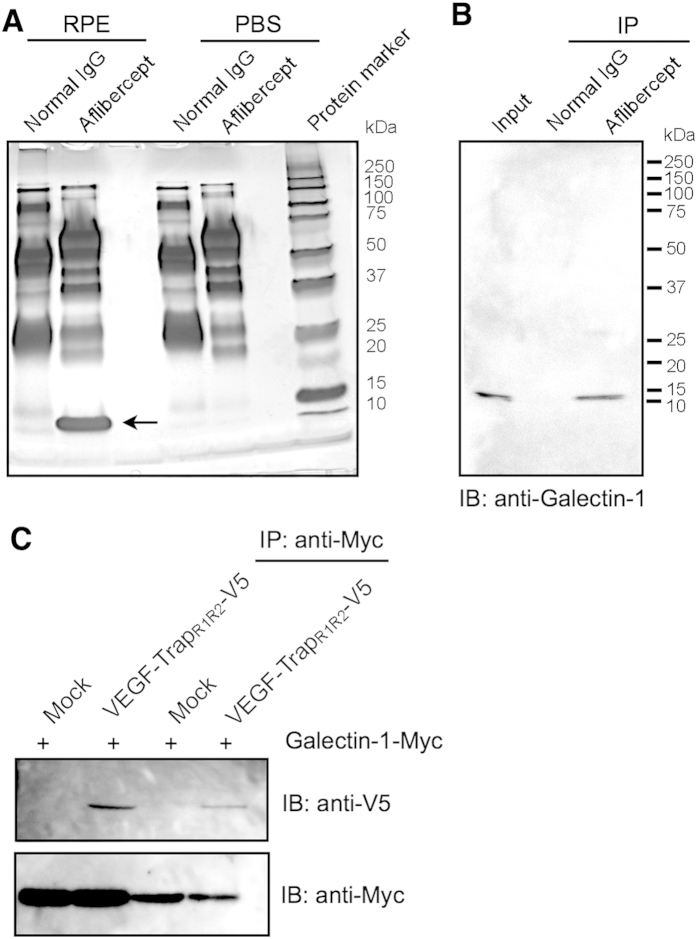
Molecular binding of aflibercept with galectin-1. (**A**) Human RPE cell extracts were applied with aflibercept- or normal IgG-immobilized protein G beads. The eluted proteins were separated by SDS-PAGE and visualized with silver staining. A single protein band around 14 kDa was detected (arrow). (**B**) Co-IP of human RPE cell extracts using aflibercept was performed, followed by SDS-PAGE and immunoblot (IB) analyses for galectin-1. (**C**) Co-transfection and Co-IP. V5-tagged *VEGF-Trap*_*R1R2*_ expression plasmids were co-transfected into HEK293T cells with Myc-tagged *Galectin-1/LGASL1* expression constructs. IP was performed with anti-Myc antibody followed by detection with anti-V5 antibody.

To isolate aflibercept-interacting proteins from the human retinal pigment epithelium (RPE) cell line, we first performed immunoprecipitation (IP) using aflibercept and RPE cell extracts. The bound proteins were eluted, analyzed by electrophoresis, and detected by silver staining (Fig. 1A). A significant protein band corresponding to approximately 14 kDa was observed in the immunoprecipitates with aflibercept as compared to those with normal human IgG and PBS with aflibercept (Fig. 1A), suggesting a specific interaction between this protein and aflibercept.

This protein band (Fig. 1A) was excised from the gel and trypsinized. The resulting peptides were analyzed by mass spectrometry (MS) and searched with the Mascot program. As a result, one protein, galectin-1, was identified (Table 1). In addition, we performed these IP and MS procedures using rat retinal capillary endothelial cells (TR-iBRB2) so as to obtain the same results (Supplementary Fig. S1).

### Molecular binding of aflibercept with galectin-1

To validate the IP and MS results (Fig. 1A and Table 1), we performed co-IP experiments using RPE cell extracts as an input sample with aflibercept and immunoblotting with anti-galectin-1 antibody. In accordance with the IP/MS data (Fig. 1A), aflibercept demonstrated the precipitation of galectin-1 protein from the RPE cell extracts, while the input RPE cell extracts used as a positive control showed an immunoreactive band of the predicted size (Fig. 1B).

To further verify the molecular interaction between aflibercept and galectin-1, co-IP experiments followed by immunoblot analyses were performed with transfected HEK293T cells using antibodies against V5- or Myc-tag. We subcloned *VEGF-Trap*_*R1R2*_ and *LGALS1* cDNAs into expression vectors containing V5 and Myc, respectively, and co-transfected both of these constructs into HEK293T cells. IP with anti-Myc antibody from the co-transfected cell extracts demonstrated that galectin-1 could pull down aflibercept (Fig. 1C), further confirming the protein interaction between aflibercept and galectin-1.

### Binding affinity of aflibercept with galectin-1

**Figure 2:**
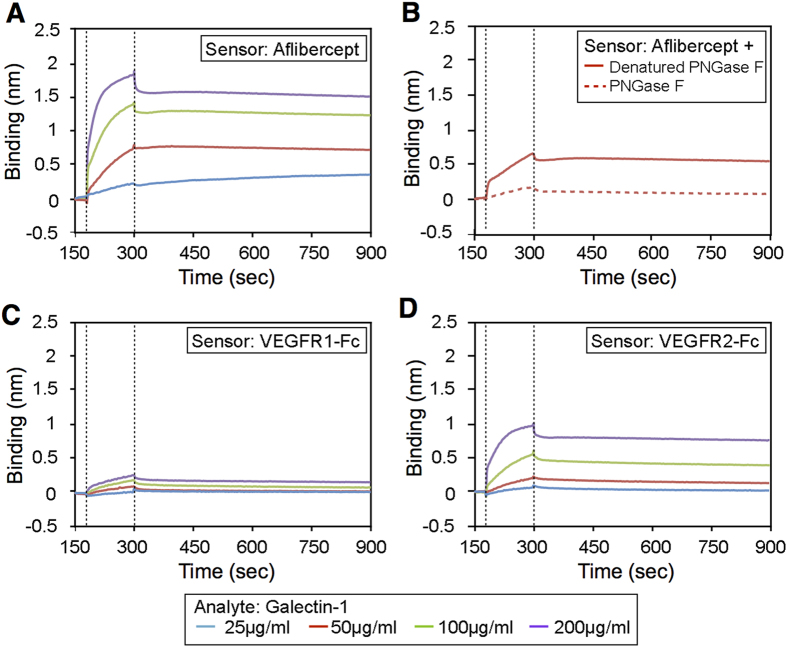
Binding affinity of aflibercept with galectin-1. (**A**) Kinetic analysis of galectin-1 binding with aflibercept using the BLItz biolayer interferometry system. Sensorgrams obtained using aflibercept-loaded biosensors incubated with different concentrations of galectin-1 as analytes. Dotted lines indicate the start of the binding (*left*) and the dissociation (*right*) phase. (**B**) Sensorgrams obtained using biosensors loaded with aflibercept with (*dotted line*) or without (*solid line*) deglycosylation (+ PNGase F or Denatured PNGase F, respectively) and incubated with galectin-1 at the concentration of 50 μg/ml. (**C**,**D**) Sensorgrams obtained using biosensors loaded with VEGFR1-Fc (**C**) or VEGFR2-Fc (**D**) and incubated with different concentrations of galectin-1.

To determine the affinity of the interaction between aflibercept and galectin-1, and to investigate the influence of stoichiometry on the interaction, sensorgrams were collected for galectin-1 using biosensors loaded with aflibercept. Analysis for the sensorgrams of Fig. 2A yielded a dissociation constant (*K*_*D*_) of 23.68 nM for the interaction of galectin-1 with aflibercept (Table 2). Recently, galectin-1 has been shown to interact with *N*-glycans on Ig domains 3, 4 and 7 of VEGFR2^18^, one of which (Ig domain 3) is utilized as the VEGFR2 region of aflibercept^16^. To test a requirement of *N*-glycosylation of aflibercept for its interaction with galectin-1, we incubated aflibercept with peptide:*N*-glycosidase F (PNGase F) to cleave *N*-linked glycans. PNGase F treated-aflibercept showed a significantly lower binding affinity than aflibercept treated with denatured PNGase F (Fig. 2B), and yielded a *K*_*D*_ of 868.7 nM for the interaction of deglycosylated aflibercept with galectin-1 (Table 2 and Supplementary Fig. S2). These results suggest that galectin-1 recognizes and binds the *N*-glycans on the VEGFR2 region of aflibercept.

Next, in order to verify the significance of the VEGFR2 region to interact with galectin-1, we measured and compared the binding affinity of galectin-1 with VEGFR1-Fc (Fig. 2C) and VEGFR2-Fc (Fig. 2D). *K*_*D*_ values for the interaction of galectin-1 with VEGFR1-Fc and VEGFR2-Fc were 3032 nM and 334.3 nM, respectively (Table 2), showing a considerably lower affinity of VEGFR1-Fc than VEGFR2-Fc with galectin-1, in consistence with the recent report^18^. The data also confirmed the robust binding of aflibercept to galectin-1 with higher affinity compared to VEGFR1-Fc and VEGFR2-Fc.

### Induction of retinal *Galectin-1/LGALS1* mRNA

**Figure 3:**
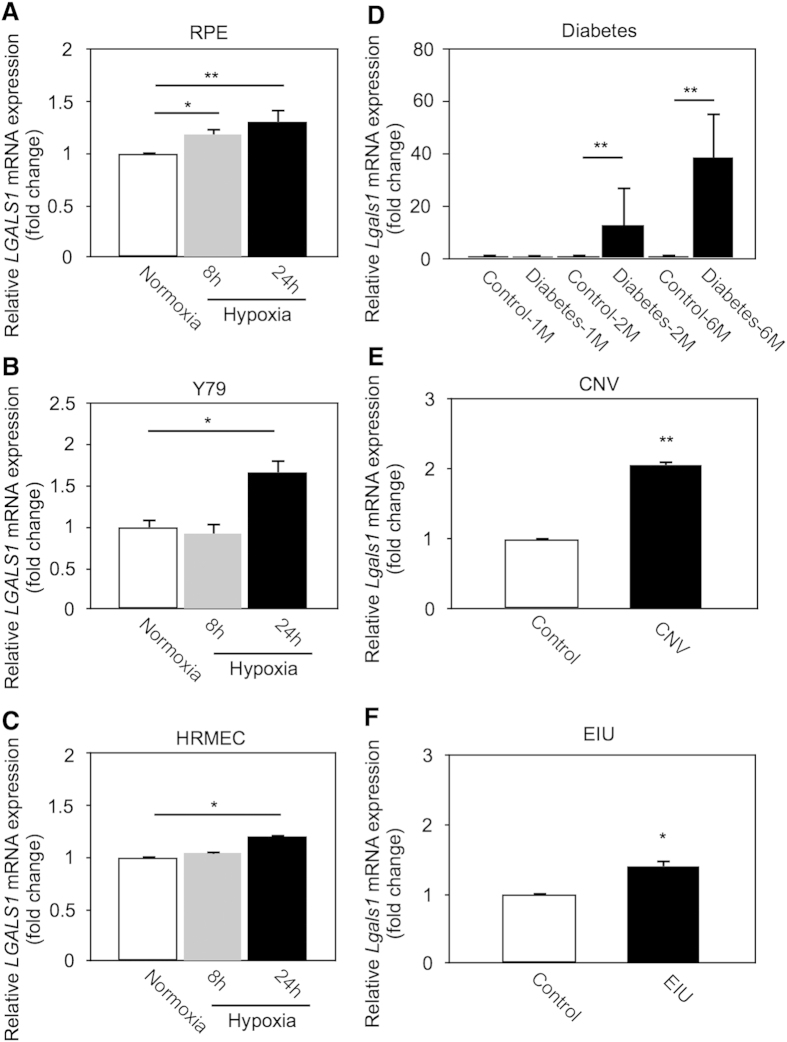
Induction of retinal *Galectin-1/LGALS1* mRNA *in vitro* and *in vivo*. (**A**–**C**) *LGALS1* mRNA expression in hypoxia (1% O_2_)-stimulated cell culture using RPE (**A**), Y79 (**B**) and HRMEC (**C**). (**D–F**) *Lgals1* mRNA expression in retinal tissues from disease model mice with streptozotocin-induced diabetes (**D**), CNV (**E**) and EIU (**F**). *p < 0.05, **p < 0.01 (n = 6 per group).

Galectin-1 was reported to be expressed physiologically and pathologically in various regions of the mammalian retina, including the RPE, external limiting membrane, and outer plexiform layer^19^,^20^. Hypoxia-inducible factor (HIF)-1α protein, an oxygen-sensitive subunit of HIF-1 that is a master factor for cellular response to hypoxia, regulates *LGALS1/Galectin-1* mRNA in cancer cells^21^. To investigate the effect of hypoxic stimulation on *LGALS1* mRNA expression in human retinal cell lines, we checked *LGALS1* mRNA levels using real-time PCR. Hypoxia led to significant upregulation of *LGALS1* gene expression in various human cell lines including RPE (Fig. 3A; 8 h, fold change = 1.19; 24 h, fold change = 1.31), Y79 (Fig. 3B; 8 h, fold change = 0.92; 24 h, fold change = 1.67), and human retinal microvascular endothelial cells (HRMEC) (Fig. 3C; 8 h, fold change = 1.05; 24 h, fold change = 1.21).

Next, we checked the induction of murine *Lgals1* mRNA in various eye disease models; streptozotocin-induced diabetes, laser-induced CNV, and endotoxin-induced uveitis (EIU); all of which were shown to involve the activation of the HIF-1 pathway^22^,^23^,^24^. *In vivo, Lgals1* mRNA levels were significantly upregulated in the retina (diabetes at months 1, 2 and 6; EIU at day 1) and the RPE-choroid complex (CNV at day 4), compared to controls (Fig. 3D; Diabetes-1M, fold change = 0.89; Diabetes-2M, fold change = 12.83; Diabetes-6M, fold change = 38.62. Fig. 3E; CNV, fold change = 2.06. Fig. 3F; EIU, fold change = 1.41).

### Localization of galectin-1 in PDR fibrovascular tissues

**Figure 4:**
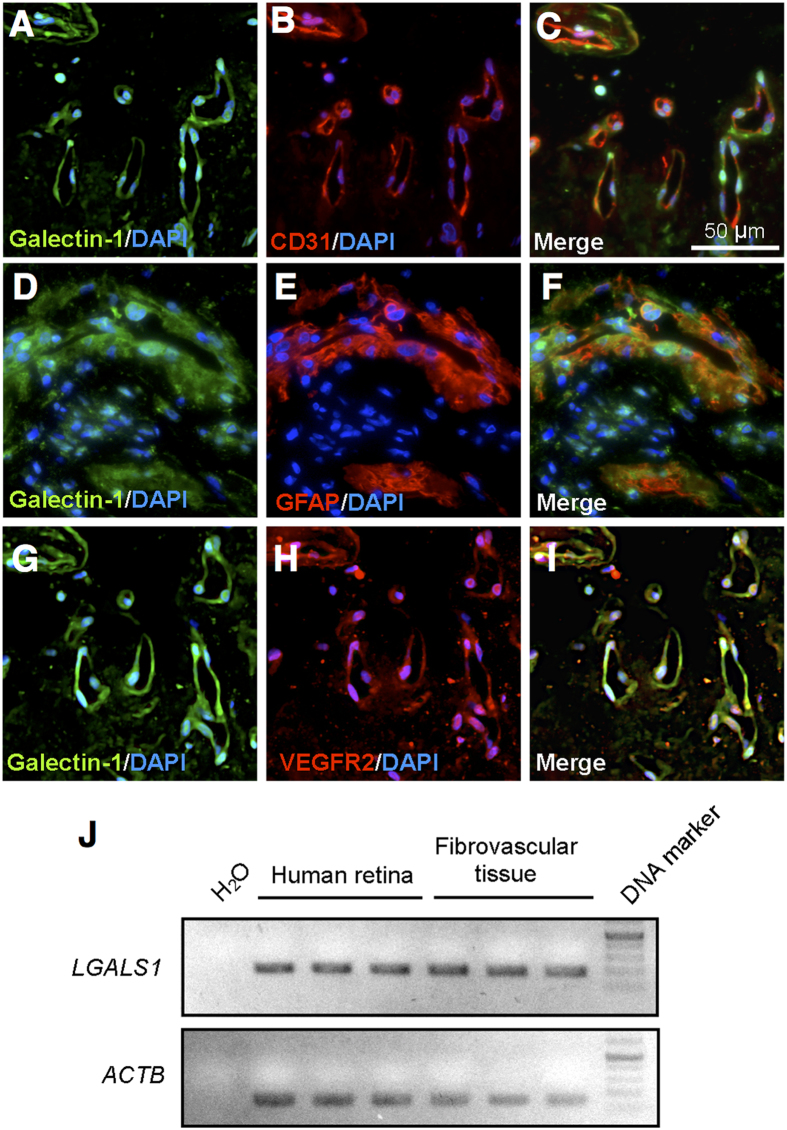
Localization of galectin-1 in human PDR fibrovascular tissues. (**A**–**C)** Double labeling of galectin-1 (*green*), CD31 (*red*) and DAPI (*blue*) in PDR fibrovascular tissues. (**D**–**F**) Double labeling of galectin-1 (*green*), GFAP (*red*) and DAPI (*blue*) in PDR fibrovascular tissues. (**G–I**) Double labeling of galectin-1 (*green*), VEGFR2 (*red*) and DAPI (*blue*) in PDR fibrovascular tissues. Scale bar = 30 μm. (**J**) End-point PCR analysis. Gene expression of *LGALS1* in healthy human retinas and PDR fibrovascular tissues.

To examine the tissue localization and expression of galectin-1, we carried out immunofluorescence and RT-PCR for fibrovascular tissues surgically excised from human PDR eyes. Double-staining experiments demonstrated co-localization of galectin-1 with CD31 (Fig. 4A–C) and GFAP (Fig. 4D–F), showing its ubiquitous distribution to neovascular endothelial cells and glial cells, respectively, both of which were the major cellular components in the fibrovascular tissues. Importantly, galectin-1 co-localized with VEGFR2 in the new vessels (Fig. 4G–I), suggesting the VEGFR2-mediated pathogenic role of galection-1 in PDR as well as in tumor angiogenesis^18^,^21^. We also confirmed mRNA expression of *LGALS1* in healthy adult retinas and the fibrovascular tissues (Fig. 4J).

### Elevation of galectin-1 protein levels in PDR vitreous fluids

**Figure 5:**
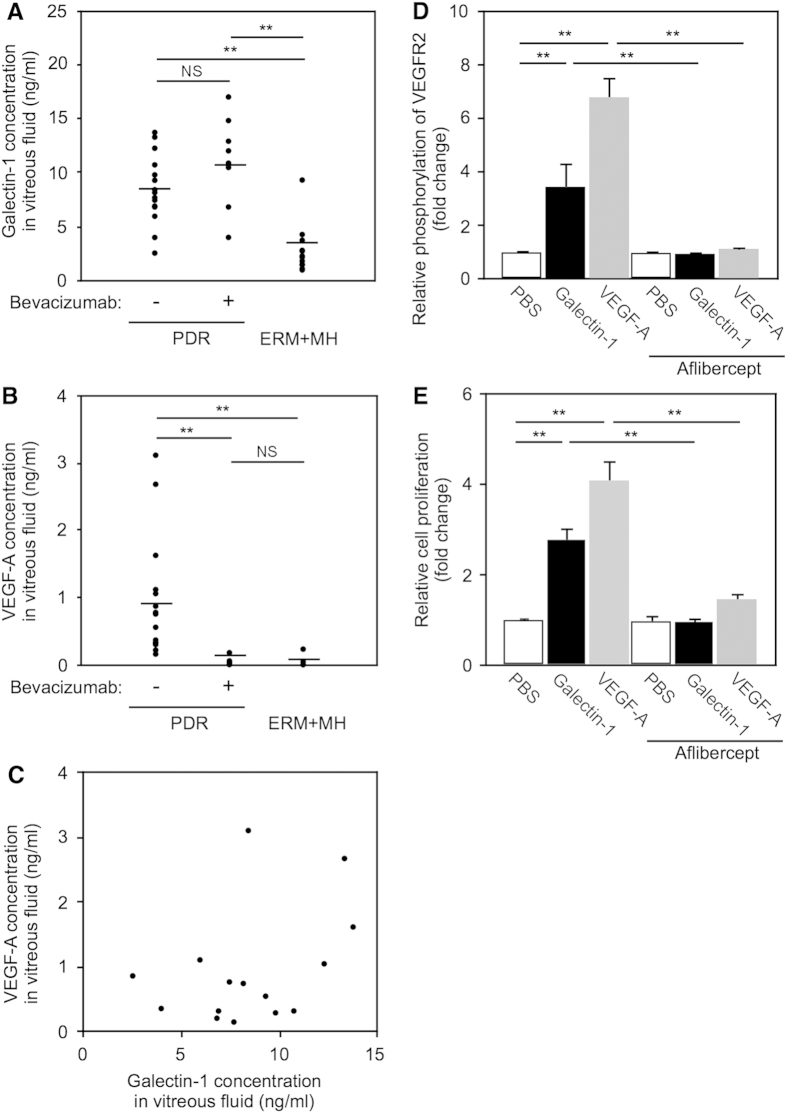
Elevation of galectin-1 protein levels in human PDR vitreous fluids and suppression of galectin-1-mediated VEGFR2 activation by aflibercept in HRMEC. (**A**) Protein levels of galectin-1 in PDR eyes with (n = 8) or without (n = 15) bevacizumab pretreatment and in control (ERM + MH) eyes (n = 12). Black symbols indicate individual samples in each group with a bar showing the average. **p < 0.01. (**B**) Protein levels of VEGF-A in PDR eyes with or without bevacizumab pretreatment and in control eyes. (**C**) No correlation (*p* = 0.137, *r*^*2*^ = 0.150) between galectin-1 and VEGF-A in PDR eyes without bevacizumab pretreatment. (**D**) VEGFR2 phosphorylation induced by galectin-1 as well as VEGF-A, both of which were significantly suppressed by aflibercept. Data are shown as relative values compared to PBS (n = 5 per group). (**E**) HRMEC proliferation induced by galectin-1 and VEGF-A, both of which were significantly suppressed by aflibercept. Data are shown as relative values compared to PBS (n = 8 per group).

To investigate the production of galectin-1 in PDR eyes, we performed ELISA experiments to measure galectin-1 protein levels in vitreous fluids aspirated from PDR and non-diabetic control eyes with idiopathic epiretinal membrane (ERM) and macular hole (MH). We divided PDR patients into two groups with or without intravitreal bevacizumab (anti-VEGF-A monoclonal antibody) injection 2-3 days before surgery (bevacizumab-treated, 8 eyes; non-treated, 15 eyes). Galectin-1 levels were significantly elevated in the vitreous fluids of PDR eyes (Bevacizumab −, 8.46 ± 0.82 ng/ml; Bevacizumab +, 11.12 ± 1.48 ng/ml) compared with controls (2.82 ± 0.65 ng/ml) (Fig. 5A). Notably, there was no significant difference in galectin-1 levels between eyes with or without bevacizumab pretreatment, in stark contrast to the following data on VEGF-A.

Intraocular levels of VEGF-A, a key molecule responsible for angiogenesis, were shown to be increased in PDR eyes^4^. To examine the relationship between galectin-1 and VEGF-A, we checked VEGF-A protein levels in the PDR vitreous samples. VEGF-A protein levels in PDR eyes receiving no bevacizumab (0.948 ± 0.230 ng/ml) were significantly higher than those of bevacizumab-treated PDR eyes (0.041 ± 0.021 ng/ml) and controls (0.023 ± 0.019 ng/ml) (Fig. 5B). Interestingly, in PDR eyes without bevacizumab pretreatment, intravitreal protein levels of VEGF-A were not correlated with those of galectin-1 (Fig. 5C), suggesting that these two molecules were independently regulated.

### Suppression of galectin-1-mediated VEGFR2 activation by aflibercept

Galectin-1 binding to VEGFR2 has recently been shown to activate the VEGFR2 signaling pathway^18^,^25^. To determine the effect of aflibercept on galectin-1-mediated VEGFR2 activation, we evaluated the relative phosphorylation of VEGFR2 *in vitro* by ELISA. Administration of galectin-1 as well as VEGF-A to HRMEC significantly increased VEGFR2 phosphorylation compared with controls (Galectin-1, fold change = 3.45; VEGF-A, fold change = 6.80), both of which were remarkably suppressed by aflibercept (Galectin-1 + aflibercept, fold change = 0.95; VEGF-A + aflibercept, fold change = 1.13; Control + aflibercept, fold change = 0.98) (Fig. 5D).

Phosphorylation of VEGFR2 in vascular endothelial cells causes its growth signal activation, largely via the second messenger phospholipase C-γ and subsequent mitogen-activated protein kinase cascades, leading to cell proliferation and angiogenesis^2^,^3^. To investigate the effect of aflibercept on galectin-1-induced angiogenesis, we performed proliferation assay using HRMEC. In accordance with a previous report using human umbilical vein endothelial cells^25^, administration of galectin-1 as well as VEGF-A to HRMEC significantly stimulated cell proliferation compared with controls (Galectin-1, fold change = 2.77; VEGF-A, fold change = 4.09), both of which were notably inhibited by pretreatment with aflibercept (Galectin-1 + aflibercept, fold change = 0.96; VEGF-A + aflibercept, fold change = 1.46; Control + aflibercept, fold change = 0.97) (Fig. 5E). These findings demonstrated the capability of aflibercept for blocking the angiogenic function of galectin-1 as a VEGFR2 ligand.

## Discussion

In various physiological and pathological conditions, cell-surface glycans are remodeled by the sequential enzymatic action of glycosyltransferases and glycosidases. Glycans can regulate molecular interactions that involve both homotypic and heterotypic bindings, and have multiple principal biological functions in protein maturation/turnover, cell adhesion/trafficking, and receptor binding/activation^26^,^27^. In the present study, aflibercept, the VEGFR1/VEGFR2-fused glycan-modified protein, was shown to interact with galectin-1 (Fig. 1), and its binding ability to galectin-1 was abolished by deglycosylation with a specific *N*-glycosidase (Fig. 2). There are some possible explanations for the currently observed higher affinity of galectin-1 with aflibercept than with VEGFR2. Firstly, different host cells used for generating these recombinant proteins, *i.e.*, CHO (rodent ovary) cells for aflibercept and HEK293 (human kidney) cells for VEGFR2-Fc, are likely to individually change carbohydrate structures on their Ig domain 3. Another possibility is that protein surface accessibilities may be altered because VEGFR2-Fc used here is comprised of the entire extracellular domain of VEGFR2 (Met1-Glu764) in contrast to the limited region (Ig domain 3, Leu105-Lys205) contained in aflibercept.

Under pathological conditions such as hypoxia responsible for various ocular disorders, *LGALS1* (*Lgals1*) mRNA expression levels were upregulated in human retinal cell lines and the murine retinal tissues (Fig. 3). Furthermore, galectin-1 was expressed and co-localized with VEGFR2 in neovascular endothelial cells in fibrovascular tissues surgically excised from eyes of patients with PDR (Fig. 4). Importantly, vitreous aspirates from PDR eyes showed protein levels of galectin-1 higher than those from controls, which was not correlated with VEGF-A levels also increased in PDR eyes (Fig. 5). Preoperative treatment with an anti-VEGF-A antibody bavacizumab failed to reduce galection-1 levels in PDR eyes, whereas aflibercept administration to human retinal endothelial cells significantly suppressed galectin-1-stimulated VEGFR2 activation (Fig. 5). Our current data provided a novel insight into anti-VEGF-A therapeutic strategies in terms that aflibercept traps galectin-1, a VEGF-A-independent VEGFR2 ligand associated with the pathogenesis of PDR as well as various retinal disease models.

Galectins, a galactoside-binding lectin protein family, have an evolutionally conserved carbohydrate-recognition domain capable of interacting with ß-galactosides, particularly *N*-acetyllactosamine-containing glycans. Galectins bind to cell surface glycol-conjugated proteins or lipids, and modify cellular functions without having specific receptors like cytokines^28^. Various biological functions of galectins include homeostasis, apoptosis, and pathological conditions such as inflammation, cancer and diabetes^29^,^30^. Several transcription factors such as HIF-1α, nuclear factor-κB and activator protein-1 are known to regulate *LGALS1/Galectin-1* gene expression^21^,^31^. Galectin-1 plays multifaceted roles in cell adhesion/proliferation, angiogenesis and immunosuppression, targeting not only a variety of cancer cells, but also vascular endothelial cells and regulatory T cells^21^,^32^. Suppression of *LGALS1* expression by RNA interference was shown to increase sensitivity to anti-cancer drugs^33^. In mammalian eyes, galectin-1 was found in various regions including the RPE, photoreceptor inner segment, external limiting membrane, outer and inner plexiform layers, nerve fiber layer, and choroidal vessels^19^,^20^. In human specimens, galectin-1 expression co-localized to the migrated RPE cells in fibrous tissues surgically removed from eyes with proliferative vitreoretinopathy, and *in vitro* stimulation by hepatocyte growth factor caused a migratory RPE phenotype via elevation of galectin-1 levels, mimicking the pathogenesis of proliferative vitreoretinopathy^19^.

Recently, galectin-1 was reported to play a crucial role in promoting angiogenesis in anti-VEGF-A refractory tumors^18^. Galectin-1 was shown to interact with the *N*-glycans on Ig domains 3, 4 and 7 of VEGFR2, causing increased phosphorylation of VEGFR2 and activation of its downstream signaling pathways in endothelial cells. Anti-VEGF-A refractory tumors enhanced galectin-1 secretion together with its increased binding to neovascular endothelial cells due to altered glycosylation patterns (i.e., decreased α2,6-linked sialic acid) on VEGFR2, leading to galectin-1-driven angiogenesis and tumor progression. More recently, we have shown a significant increase in sialylated *N*-glycans in eyes of patients with PDR and a relative decrease in α2,6-linked sialic acid in glucose-stimulated human retinal endothelial cells^34^. In concert with the currently observed elevation of galectin-1 in PDR eyes (Fig. 5), these recent reports suggest a potential role of altered *N*-glycan profiles in the angiogenic activity in PDR as well as cancer.

Intravitreal injection of VEGF-A blockers is a first-line therapy for several ocular disorders such as wet AMD and diabetic macular edema; however, the anti-VEGF-A strategy is not necessarily effective for all patients. Despite the excellent results of numerous prospective clinical trials, 10 to 30% of patients have been shown to be non-responders for anti-VEGF-A drugs^35^,^36^. Although the retrospective design and the small number of cases constitute limitations, several reports have suggested the efficacy of switching to aflibercept in patients with wet AMD who were primarily or secondarily refractory to other anti-VEGF-A agents^37^,^38^,^39^. In a prospective head-to-head clinical trial, aflibercept has been shown to be more effective in improving vision than other two blockers for patients with diabetic macular edema at relatively low levels of initial visual acuity^40^.

Several biological properties of aflibercept may explain the theoretical rationale of these efficacious results suggesting its superiority. Aflibercept binds to VEGF-A with much higher affinity than other VEGF-A blockers, and a low potential for immunogenicity due to its human-based sequence allows for a slow clearance by conferring a long half-life^16^. Moreover, aflibercept is distinguished from other anti-VEGF-A drugs by its ability to bind other VEGF family members such as placental growth factor^17^, which was also strongly correlated with angiogenic stages of diabetic retinopathy^41^. Lastly, our present findings revealed an additional anti-angiogenic role of aflibercept in neutralizing galectin-1, a non-VEGF family angiogenic factor associated with human diabetic retinopathy.

## Methods

### Cell lines and animals

Human RPE (hTERT-RPE1), retinoblastoma (Y79) and HEK293T cells were obtained from American Type Culture Collection (Manassas, VA). Rat retinal endothelial cells (TR-iBRB2) were provided from Fact Inc. (Sendai, Japan)^42^. HRMEC, CS-C medium, a growth medium optimized for HRMEC, and CultureBoost were purchased from Cell Systems (Kirkland, WA). For hypoxic exposure, cells were cultured in a gas mixture composed of 1% O_2_, 5% CO_2_, and 94% N_2_. For cell stimulation, after being serum-deprived, cells were treated with 10 μg/ml galectin-1 or 5 ng/ml VEGF-A (R&D systems, Minneapolis, MN) at 37 °C for 10 min with or without 7.5 μg/ml aflibercept (Santen Pharmaceutical Co. Ltd, Osaka, Japan) to measure VEGFR2 phosphorylation. For cell proliferation, cells were treated in the same way but for incubation time of 48 h.

C57BL/6J mice were obtained from CLEA Japan (Tokyo, Japan). All animal experiments were conducted in accordance with the ARVO Statement for the Use of Animals in Ophthalmic and Vision Research, and approved by the Ethics Review Committee for Animal Experimentation of Hokkaido University. Procedures for murine models of streptozotocin-induced diabetes, laser-induced CNV and EIU were described in our previous reports^43^,^44^,^45^.

### Immunoprecipitation

Cells were homogenized in TBS containing 1% NP-40 and protease inhibitors (Roche Applied Science, Indianapolis, IN). After pre-incubation of samples with Protein G beads (Life Technologies, Carlsbad, CA) for 1 h at 4 °C, antibodies were added and left overnight at 4 °C with gentle mixing. The beads were washed three times with lysis buffer, and suspended in SDS sample buffer.

### Nanoscale liquid chromatography with tandem mass spectrometry (nanoLC-MS/MS)

Immunoprecipitated samples were resolved on a 4–15% Mini-PROTEAN TGX gels (Bio-Rad, Hercules, CA) and stained using a Mass silver stain kit (Wako Pure Chemical Industries, Osaka, Japan). Gel slippage was reduced by 100 mM of dithiothreitol and alkylated by 100 mM idoacetamide. After washing, gels were incubated with trypsin overnight at 30 °C. Recovered peptides were desalted by Ziptip c18 (Millipore, Billerica, MA). Samples were analyzed by nanoLC-MS/MS systems (DiNa HPLC system, KYA Technologies, Tokyo, Japan; QSTAR XL, Life Technologies). Mass data acquisitions were piloted by Mascot software for matching proteins in the NCBI database.

### Immunoblot analyses

Cell extracts were lysed in SDS buffer and a protease inhibitor cocktail (Roche Applied Science). Proteins were resolved by 10% SDS-PAGE (polyacrylamide gel electrophoresis) and transferred to PVDF (polyvinylidene difluoride) membrane (Millipore) by electroblotting. Membranes were blocked in TBS containing 5% skim milk, and incubated with the following primary antibodies: anti-Galectin-1 (Abcam, Cambridge, MA) antibody, anti-V5 antibody (Life Technologies) and anti-Myc antibody (Medical & Biological Laboratories, Nagoya, Japan). The secondary antibodies for chemoluminescence detection were peroxidase-conjugated anti-mouse or anti-rabbit IgGs (Jackson ImmunoResearch Laboratories, West Grove, PA). Signal was obtained by enhanced chemoluminescence (Western Lightning Ultra, Perkin Elmer, Waltham, MA).

### Plasmid constructions

Human *LGALS1/Galectin-1* cDNA (GenBank No. NM_002305) was obtained from DNASU Plasmid Repository (Tempe, AZ) and subcloned into pCMV-Tag 3B vector (Agilent Technologies) with Myc tag and pGEX4T-2 vector (GE Healthcare, Piscataway, NJ) for glutathione S-transferase (GST) fusion. *VEGF-Trap*_*R1R2*_ (corresponding to aflibercept) cDNA^16^ was generated as a synthetic gene by IDT (Coralville, IA), and subcloned into the pcDNA6.2/V5-DEST vector (Life Technologies) with V5 tag. The empty pcDNA6.2/V5-DEST vector was used as a Mock transfection control. The GST-fused galectin-1 protein was expressed in an *Escherichia coli* strain Rosetta-gami 2 (DE3) (Novagen, Madison, WI), and purified through binding to Glutathione Sepharose (GE Healthcare). All constructs were sequence-verified before use.

### Biolayer interferometry

Protein binding affinities were quantified by biolayer interferometry using the BLItz instrument (ForteBio, Menlo Park, CA). Aflibercept, VEGFR1-Fc and VEGFR2-Fc (Sino Biological Inc., Beijing, China) proteins were immobilized on Protein A-coated biosensors, and equilibrated in buffer to establish a stable baseline as sensors, then dipped into various concentrations of GST-galectin-1 as analytes. All protein-binding experiments were done in PBS. Control values, measured using empty (no protein-loaded) sensors, were subtracted from experimental values before data processing. Preliminary binding experiments with GST protein using empty sensors and aflibercept-loaded sensors and with GST-galectin-1 using normal human IgG-loaded sensors yielded similar almost zero values indicating no specific binding (data not shown). For deglycosylation, aflibercept was incubated with PNGase F (New England Biolabs, Ipswich, MA) according to the manufacturer’s instructions. As a negative control, PNGase F was inactivated by heat denaturation at 100°C for 10 min. The equilibrium dissociation constant (*K*_*D*_) together with association (*k*_*a*_) and dissociation (*k*_*d*_) rate constants were calculated using BLItz Pro software.

### RT-PCR and real-time PCR

Total RNA isolation and reverse transcription were performed from cells using SuperPrep Cell Lysis & RT Kit for qPCR (TOYOBO, Tokyo, Japan) and from tissues using TRIzol (Life Technologies) and GoScrip Reverse Transcriptase (Promega, Madison, WI) with oligo dT(20) primers, as previously described^44^. The following primers for genes were used: human *LGALS1* (galectin-1; forward 5′- CGC TAA GAG CTT CGT GCT GAA C-3′, reverse 5′-CAC ACC TCT GCA ACA CTT CCA G -3′), human *ACTB* (ß-actin; forward 5′-CTG GAA CGG TGA AGG TGA CA -3′, reverse 5′-AAG GGA CTT CCT GTA ACA ATG CA -3′), mouse *Lgals1* (forward 5′-TCC CCG AAC TTT GAG ACA TTC -3′, reverse 5′-GTC TCA GGA ATC TCT TCG CTT C -3′, probe 5′-ACC AGA CCA CAG GCC ATG ATT GAA -3′), and mouse *Gapdh* (glyceraldehyde-3-phosphate dehydrogenase; forward 5′-AGG TCG GTG TGA ACG GAT TTG -3′, reverse 5′-TGT AGA CCA TGT AGT TGA GGT CA -3′).

### Human surgical samples

Vitreous fluids were collected from 23 eyes of 23 patients (11 males and 12 females, average age = 55.0 ± 2.1 years) with PDR. Undiluted vitreous samples were collected at the start of pars plana vitrectomy using the transconjunctival sutureless vitrectomy system (23G or 25G) and were frozen rapidly at −80 °C. Control vitreous samples were obtained from 12 eyes of 12 age-matched, non-diabetic patients (4 males and 8 females, average age = 60.4 ± 1.9 years) with idiopathic macular diseases including ERM and MH. During surgery, 5 fibrovascular tissues were excised from PDR eyes and used for immunohistochemistry, and additional 3 fibrovascular tissues were processed for gene expression analyses. This study was conducted in accordance with the tenets of the Declaration of Helsinki and after receiving approval from the institutional review board of Hokkaido University Hospital. All patients gave written informed consent after our explanation of the purpose and procedures of this study. We obtained 3 human (healthy adults) retina cDNAs as a kind gift from Dr. Anand Swaroop (National Eye Institute, Bethesda, MD).

### Immunofluorescence microscopy

Paraffin sections of fibrovascular tissues were deparaffinized and hydrated through exposure with xylene and graded alcohols followed by water. As a pretreatment, microwave-based antigen retrieval was performed in 10 mM citrate buffer (pH 6). Sections were probed with the following primary antibodies: anti-Galectin-1 (Abcam), anti-VEGFR2 (R&D systems, Minneapolis, MN), anti-CD31 (DAKO, Tokyo, Japan), and anti-GFAP (glial fibrillary acidic protein; Leica, Exton, PA) antibodies. The secondary antibodies for fluorescent detection were AlexaFluor 488 and 546 (Life Technologies). Nuclei were counterstained with DAPI (diamidino-2-phenylindole), and sections were visualized under a Biorevo microscope (Keyence, Tokyo, Japan).

### ELISA

The protein levels of galectin-1 and VEGF-A in vitreous fluids and those of VEGFR2 and phosphorylated VEGFR2 *in vitro* were determined with human Galectin-1, VEGF-A, VEGFR2 and Phospho-VEGFR2 ELISA kits (R&D systems) per the manufacturer’s instructions. Levels of phosphorylated VEGFR2 were normalized by VEGFR2 protein concentrations. The optical density was determined using a micro plate reader (Sunrise, TECAN, Männedorf, Switzerland).

### Cell proliferation assay

RealTime-Glo MT cell viability assay (Promega) was used to quantify viable cells, according to the manufacturer’s instructions. The luminescent signal was determined using Infinite 200 PRO (TECAN).

### Statistical analyses

All results were expressed as mean ± SEM. The values were processed for statistical analyses (Student’s t-test and Spearman rank correlation). Differences between the means were considered statistically significant when the *P* values were < 0.05.

## Additional Information

**How to cite this article**: Kanda, A. *et al.* Aflibercept Traps Galectin-1, an Angiogenic Factor Associated with Diabetic Retinopathy. *Sci. Rep.* **5**, 17946; doi: 10.1038/srep17946 (2015).

## Supplementary Material

Supplementary Information

## Figures and Tables

**Table 1:** Summary of identified aflibercept-interacting protein.

Identified protein	Gene name	Molecular mass (Da)	No. of peptides	Score	Sequence coverage (%)	Calculated pI value	*p* value	Accession number
Galectin-1	*LGALS1*	14,716	11	184	77	5.34	<0.05	P09382

**Table 2:** Equilibrium dissociation constant (*K*_*D*_) and kinetic constants (*k*_*a*_ and *k*_*d*_).

Sensor	*K*_*D*_	*k*_*a*_(1/Ms)	*k*_*d*_(1/s)
Aflibercept	23.68 nM	1.57E+4 ± 0.03E+4	3.71E-4 ± 0.32E-4
PNGase F-treated aflibercept	867.8 nM	6.12E+3 ± 0.28E+3	5.31E-3 ± 0.22E-3
VEGFR1-Fc	3032 nM	2.04E+3 ± 0.34E+3	6.18E-3 ± 0.27E-3
VEGFR2-Fc	334.3 nM	9.51E+3 ± 0.34E+3	3.18E-3 ± 0.15E-3
