# Supplementary material for: Aflibercept Traps Galectin-1, an Angiogenic Factor Associated with Diabetic Retinopathy
Source: Sci Rep. 2015 Dec 9;5:17946. doi: 10.1038/srep17946 (PMC4673700; doi:10.1038/srep17946)
Supplement: Supplementary Information [file srep17946-s1.pdf]

Aflibercept Traps Galectin-1, an Angiogenic Factor Associated with Diabetic Retinopathy

Atsuhiro Kanda, Kousuke Noda, Wataru Saito and Susumu Ishida

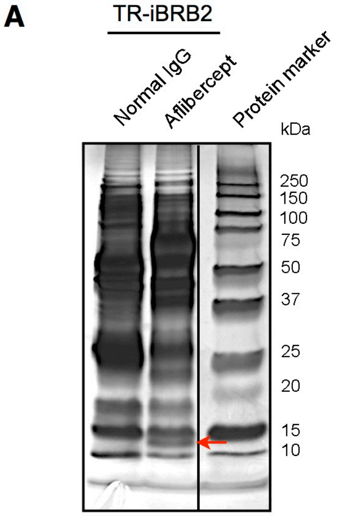

B

**Identified protein**	**Gene name**	**Molecular mass (Da)**	**No. of peptides**	**Score**	**Sequence coverage (%)**	**Calculated pI value**	***p* value**	**Accession number**
Galectin-1	*Lgals1*	14,857	16	249	80	5.14	< 0.05	P11762

Supplementary Figure S1. Molecular binding of aflibercept with galectin-1.

Rat retinal endothelial cell (TR-iBRB2) extracts were applied with aflibercept- or normal IgG-immobilized protein G beads. **A,** The eluted proteins were separated by SDS-PAGE and visualized with silver staining. A single protein band around 14 kDa was detected (red arrow). **B,** Summary of identified aflibercept-interacting protein.

Aflibercept Traps Galectin-1, an Angiogenic Factor Associated with Diabetic Retinopathy

Atsuhiro Kanda, Kousuke Noda, Wataru Saito and Susumu Ishida

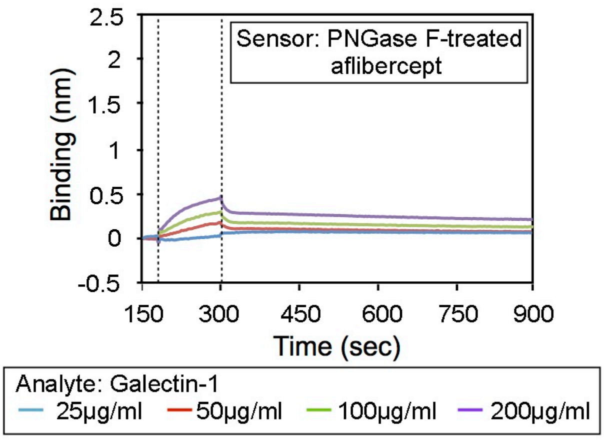

Supplementary Figure S2. Binding affinity of deglycosylated aflibercept with galectin-1. Sensorgrams obtained using biosensors loaded with PNGase F-treated aflibercept and incubated with different concentrations of galectin-1.
